# Trajectories of Controller Therapy Use Before and After Asthma-Related Hospitalization in Children and Adults: Population-Based Retrospective Cohort Study

**DOI:** 10.2196/50085

**Published:** 2023-09-26

**Authors:** Manon Belhassen, Maeva Nolin, Flore Jacoud, Claire Marant Micallef, Eric Van Ganse

**Affiliations:** 1 PELyon Lyon France; 2 Research on Healthcare Performance RESHAPE INSERM U1290 Université Claude Bernard Lyon 1 Lyon France; 3 Respiratory Medicine Croix Rousse University Hospital Lyon France

**Keywords:** asthma, hospitalization, inhaled corticosteroids, trajectories, quality of care, clustering

## Abstract

**Background:**

Inappropriate use of inhaled corticosteroids (ICSs) for asthma impairs control and may cause exacerbation, including asthma-related hospitalization (ARH). In prospective studies, ICS use peaked around ARH, but information on routine care use is limited. Since ARH is a major outcome, controller therapy use in routine care before and after ARH should be documented.

**Objective:**

This study aimed to distinguish ICS use typologies (trajectories) before and after ARH, and assess their relationships with sociodemographic, disease, and health care characteristics.

**Methods:**

A retrospective cohort study was performed using a 1% random sample of the French claims database. All patients hospitalized for asthma between January 01, 2013, and December 31, 2015, were classified as either children (aged 1-10 years) or teens/adults (aged ≥11 years). Health care resource use was assessed between 24 and 12 months before ARH. ICS use was computed with the Continuous Measures of Medication Acquisition-7 (CMA7) for the 4 quarters before and after ARH. Initially, the overall impact of hospitalization on the CMA7 value was studied using a segmented regression analysis in both children and teens/adults. Then, group-based trajectory modeling differentiated the groups with similar ICS use. We tested different models having 2 to 5 distinct trajectory groups before selecting the most appropriate trajectory form. We finally selected the model with the lowest Bayesian Information Criterion, the highest proportion of patients in each group, and the maximum estimated probability of assignment to a specific group.

**Results:**

Overall, 863 patients were included in the final study cohort, of which 447 (51.8%) were children and 416 (48.2%) were teens/adults. In children, the average CMA7 value was 12.6% at the start of the observation period, and there was no significant quarter-to-quarter change in the value (*P*=.14) before hospitalization. Immediately after hospitalization, the average CMA7 value rose by 34.9% (*P*=.001), before a significant decrease (*P*=.01) of 7.0% per quarter. In teens/adults, the average CMA7 value was 31.0% at the start, and there was no significant quarter-to-quarter change in the value (*P*=.08) before hospitalization. Immediately after hospitalization, the average CMA7 value rose by 26.9% (*P*=.002), before a significant decrease (*P*=.01) of 7.0% per quarter. We identified 3 and 5 trajectories before ARH in children and adults, respectively, and 5 after ARH for both groups. Trajectories were related to sociodemographic characteristics (particularly, markers of social deprivation) and to potentially inappropriate health care, such as medical management and choice of therapy.

**Conclusions:**

Although ARH had an overall positive impact on ICS use trajectories, the effect was often transient, and patient behaviors were heterogeneous. Along with overall trends, distinct trajectories were identified, which were related to specific patients and health care characteristics. Our data reinforce the evidence that inappropriate use of ICS paves the way for ARH.

## Introduction

Asthma is a major public health concern, and over 5% of the total population in high-income countries live with the condition [1]. Both adults and children cover the asthma demographic [2], and it not only affects quality of life but also causes significant morbidity (and in some instances mortality). It is responsible for high financial burden at both the individual and societal levels, particularly as a result of acute exacerbation [3,4].

The risk of exacerbation increases when asthma is poorly controlled, and severe exacerbation can lead to asthma-related hospitalization (ARH) [5,6]. A common cause of poor control is the inappropriate use of inhaled corticosteroids (ICSs) in monotherapy or as fixed-dose combinations (FDCs), as well as the inappropriate use of long-acting beta agonists (LABAs) in monotherapy [7,8].

In a prospective cohort study of ARH, a rapid decrease was shown in the use of ICSs and oral corticosteroids (OCSs) after hospital discharge [9]. Information on preadmission use, however, was not available. Another study clarified the overall relationship between adherence to ICSs and the occurrence of severe exacerbations, including ARH, with the use of ICSs increasing shortly before the exacerbation and further increasing afterward [10].

Nonetheless, asthmatics are likely heterogeneous in that regard, and their behaviors, particularly ICS use, may differ in real-life conditions compared with that during prospective field studies, where medication use is regularly assessed. Since ARH is a major outcome, the use of controller therapy in routine care before and after ARH should be documented. This information would incite health care professionals to adjust modifiable patient and care characteristics in order to prevent recurring exacerbations. The objectives of this study were to distinguish the typology of ICS use before and after ARH in the routine care of a representative sample of the overall asthma population, and to identify sociodemographic, disease, and health care characteristics related to ICS trajectories.

## Methods

### Data Source

The data source of this study was the Echantillon Généraliste des Bénéficiaires (EGB; General Sample of Beneficiaries), which is a 1/97th representative random sample of the French national health system (Système National des Données de Santé [SNDS]; National Health Data System) that covers more than 90% of the French population. It contains comprehensive, anonymized individual information on sociodemographic characteristics, date of death, out-of-hospital reimbursed health care expenditures (from both public and private health care providers), and hospital discharge summaries using the International Classification of Diseases 10th Revision (ICD-10) codes [11].

### Study Population

We included all patients who were hospitalized for asthma (ICD-10 codes: J45 and J46) as the singular main diagnosis between January 1, 2013, and December 31, 2015. The first recorded date of ARH was defined as the index date. We selected patients with continuous follow-up during the period between 24 months before and 12 months after the index date. Patients hospitalized for asthma-related reasons in the 12 months before the index hospitalization were excluded. Two subgroups were defined: children (1-10 years old) and teens/adults (≥11 years old).

### Study Variables

For children and teens/adults separately, we described the sociodemographic characteristics (age, gender, free access to care status as a proxy of social deprivation [given on a means-tested basis; this status supports the health expenses of more modest patients], and chronic disease status [patients who benefit from this status for a diagnosed condition are totally reimbursed for care related to that condition, ie, asthma in our study]), health care resource utilization (general practitioner visits, pediatrician visits, respiratory physician visits, emergency room [ER] visits for any cause, pulmonary function testing, and hospital admission for asthma), and initiation of asthma therapy (ICSs, LABAs, FDCs of ICSs/LABAs, leukotriene receptor antagonists [LTRAs], short-acting β2 agonists [SABAs], OCSs, and antibiotics for respiratory infections). We also described the therapeutic ratio or the “controller to total asthma medication ratio” as a proxy of the quality of asthma care. Studies have shown that patients with high therapeutic ratios (≥50%) experience fewer asthma exacerbations than those with low ratios [12-15].

### Description of ICS Use

The baseline period started at T0, 365 days before the date of the index ARH (Multimedia Appendix 1). For both children and teens/adults, ICS use (in monotherapy or FDCs) was separately assessed by computing the Continuous Measures of Medication Acquisition-7 (CMA7) for 4 quarters, before the hospital admission (from T0 to the index ARH date) and after hospital discharge. CMA7 is a standard method to assess adherence to medication, calculated as the cumulative days’ supply obtained over a series of intervals divided by the total days from the beginning to the end of the study period [16,17].

### Statistical Analysis

Descriptive statistics were used to describe the sociodemographic characteristics, health care utilization, and asthma therapy over the period between 24 and 12 months before the index ARH. The categorical variables of patient characteristics were described by providing the sample size within each modality and the relative percentages. We described the quantitative variables of health care utilization to determine the sample size and relative percentages for patients (with at least one care item) to calculate the mean and SD of the number of care items in all patients.

Initially, the overall impact of hospitalization on the CMA7 value was studied using a segmented regression analysis in both children and teens/adults [18]. Then, group-based trajectory modeling (the SAS proc traj function) was used to distinguish different groups of patients regarding the typologies of the trajectories of CMA7 for ICSs [19,20] both before and after ARH. We tested different models having 2 to 5 distinct trajectory groups before selecting the most appropriate form of the trajectories. We finally selected the model with the lowest Bayesian Information Criterion, the highest proportions of patients in each group, and the maximum estimated probability of assignment to a specific group. The chi-square test, Fisher test, and Kruskal-Wallis test were used to compare patient characteristics (including asthma therapy) across trajectory groups over the baseline period for children and teens/adults as separate groups.

The statistical analysis was performed using SAS Version 9.4 (SAS Institute).

### Ethical Considerations

This observational study was conducted on anonymized data. The Commission Nationale de l'Informatique et des Libertés (CNIL; National Informatics and Liberty Committee) provided authorization for the use of the EGB data for research purposes, and approval was obtained from the French Institute for Health Data (Institut des Données de Santé) under number 133 (granted on June 9, 2015).

## Results

### Study Population

**Figure 1:**
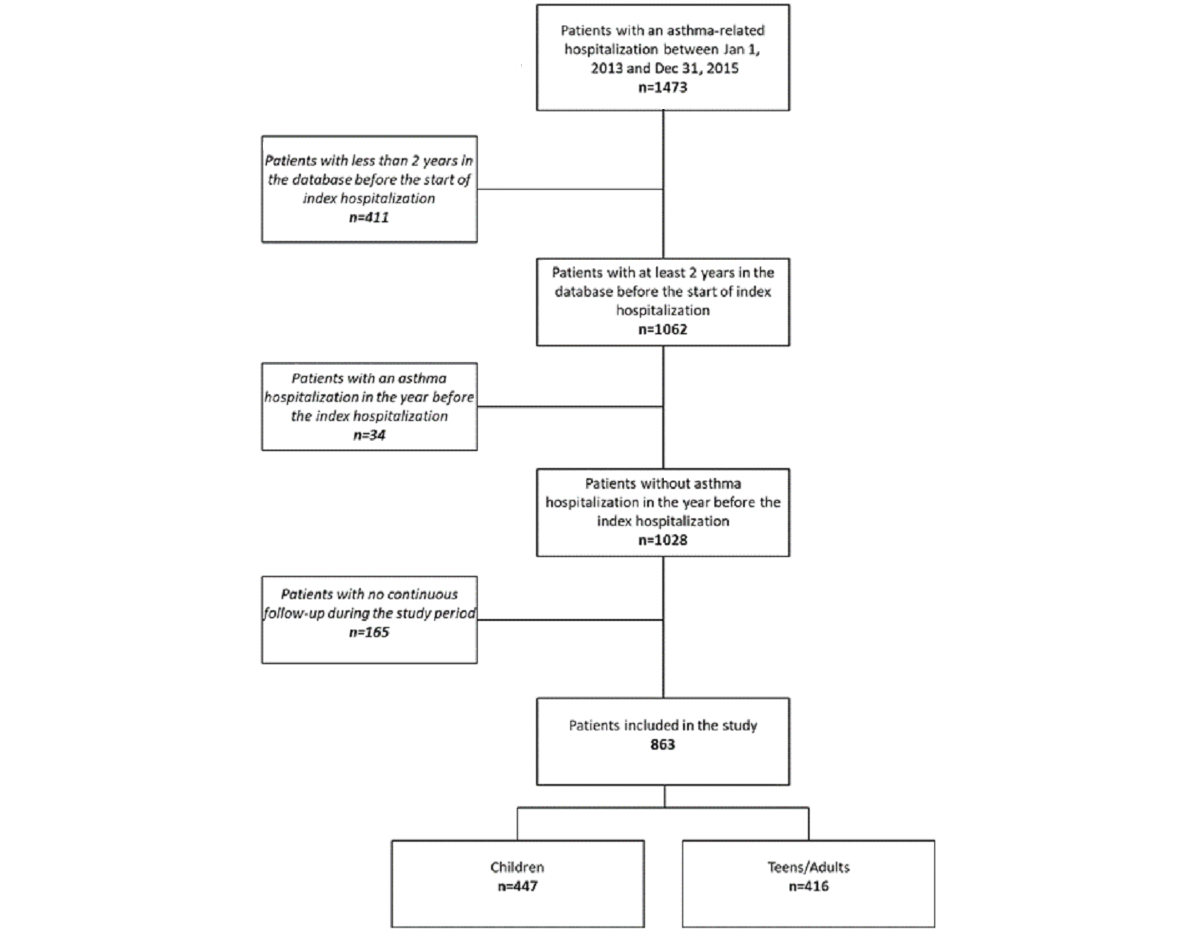
Study flowchart.

**Figure 2:**
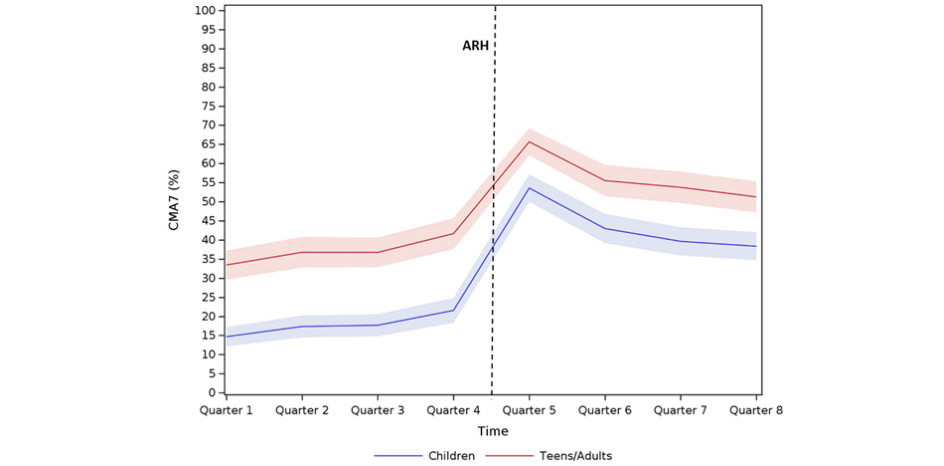
Inhaled corticosteroid use by children and teens/adults before and after asthma-related hospitalization (ARH). CMA7: Continuous Measures of Medication Acquisition-7.

In total, 1473 patients were hospitalized for asthma from January 1, 2013, to December 31, 2015. We excluded 411 patients whose claims did not span the 2 years prior to their hospitalization, 34 patients who were hospitalized for asthma in the year before the index date, and 165 patients who had no continuous follow-up. In the end, 863 patients were included, of which 447 (51.8%) were children aged 1 to 10 years and 416 (48.2%) were teens/adults aged over 11 years (Figure 1). Regarding ICS use, similar trends were observed in both the children and teens/adults (segmented regression analysis) (Figure 2).

In the children, at the start of the observation period, the average CMA7 value was 12.6%, and there was no significant quarter-to-quarter change in the value (*P*=.14) before hospitalization. Immediately after hospitalization, the average CMA7 value rose by 34.9% (*P*=.001), before a significant decrease (*P*=.01) of 7.0% per quarter.

In the teens/adults, at the start of the observation period, the average CMA7 value was 31.0%, and there was no significant quarter-to-quarter change in the value (*P*=.08) before hospitalization. Immediately after hospitalization, the average CMA7 value rose by 26.9% (*P*=.002), before a significant decrease (*P*=.01) of 7.0% per quarter.

### Population Characteristics Before ARH in the Children

At baseline, the mean age of the children was 4.2 years, and most were male (291/447, 65.1%). A quarter (112/447, 25.1%) had free access to health care. Almost half of the children (213/447, 47.7%) visited a pediatrician, whereas a quarter (115/447, 25.7%) visited a respiratory physician at least once. OCSs were dispensed to 60.9% (272/447) of the children at a mean annual dose (MAD) of 240 mg, while respiratory antibiotics were administered to 75.2% (336/447) of the children, with 4 annual dispensing events on average (Table 1). Altogether, few children (119/447, 26.6%) had high therapeutic ratios (≥0.5).

**Table 1:** Characteristics of the children at baseline (T0) overall and in the different preasthma-related hospitalization trajectories.

Characteristic				All (N=447)		No use (n=243)		Mild use (n=172)		High use (n=32)				*P* value
**Sociodemographic characteristics**														
	**Gender, n (%)**													
		Male	291 (65.1)		167 (68.7)		107 (62.2)		17 (53.1)				.13	
	Age at baseline (years), mean (SD)		4.2 (2.6)		4.1 (2.6)		4.3 (2.7)		4.8 (2.4)					
	**Age group, n (%)**												.79	
		1-5 years	320 (71.6)		177 (72.8)		120 (69.8)		23 (71.9)					
		6-10 years	127 (28.4)		66 (27.2)		52 (30.2)		9 (28.1)					
	Free access to care status, n (%)		112 (25.1)		65 (26.7)		39 (22.7)		8 (25.0)				.64	
	Chronic disease status for asthma, n (%)		12 (2.7)		1 (0.4)		8 (4.7)		3 (9.4)				<.001	
**Health care resource utilization before asthma-related hospitalization (12 months)**														
	**Pediatrician visits**												.007	
		Patients, n (%)	213 (47.7)		102 (42.0)		90 (52.3)		21 (65.6)					
		Number of visits, mean (SD)	1.9 (3.1)		1.6 (2.9)		2.1 (3.1)		3.2 (4.3)					
	**Respiratory physician visits**												.25	
		Patients, n (%)	115 (25.7)		56 (23.0)		51 (29.7)		8 (25.0)					
		Number of visits, mean (SD)	0.4 (0.9)		0.4 (0.9)		0.5 (0.8)		0.3 (0.7)					
	**Pulmonary function testing**												<.001	
		Patients, n (%)	28 (6.3)		6 (2.5)		16 (9.3)		6 (18.8)					
		Number of tests, mean (SD)	1.9 (1.5)		0.0 (0.3)		0.2 (0.8)		0.3 (0.7)					
	**Emergency room visits**												.79	
		Patients, n (%)	171 (38.3)		92 (37.9)		65 (37.8)				14 (43.8)			
		Number of visits, mean (SD)	0.7 (1.3)		0.6 (1.0)		0.8 (1.6)				0.8 (1.4)			
	**Asthma-related hospitalization**												.006	
		Patients, n (%)	27 (6.0)		7 (2.9)		18 (10.5)				2 (6.3)			
		Number of stays, mean (SD)	0.1 (0.5)		0.0 (0.2)		0.2 (0.7)				0.1 (0.6)			
**Asthma therapy**														
	**ICSs^a^**												<.001	
		Patients, n (%)	168 (37.6)		54 (22.2)		93 (54.1)				21 (65.6)			
		Number, mean (SD)	1.1 (2.4)		0.4 (1.0)		1.7 (2.6)				3.3 (5.5)			
	**FDCs^b^ of ICSs/LABAs^c^**												<.001	
		Patients, n (%)	54 (12.1)		10 (4.1)		35 (20.3)				9 (28.1)			
		Number, mean (SD)	0.4 (1.5)		0.1 (0.4)		0.7 (1.7)				1.8 (3.4)			
	**LABAs (in a separate canister)**												N/A	
		Patients, n (%)	0 (0.0)		0 (0.0)		0 (0.0)				0 (0.0)			
		Number, mean (SD)	N/A^d^		N/A		N/A				N/A			
	**LTRAs^e^**												<.001	
		Patients, n (%)	59 (13.2)		11 (4.5)		34 (19.8)				14 (43.8)			
		Number, mean (SD)	0.5 (1.6)		0.1 (0.4)		0.5 (1.4)				2.9 (4.2)			
	**SABAs^f^**												<.001	
		Patients, n (%)	233 (52.1)		91 (37.4)		114 (66.3)				28 (87.5)			
		Number, mean (SD)	1.5 (2.3)		0.8 (1.3)		2.0 (2.3)				4.3 (4.2)			
	**OCSs^g^**												<.001	
		Patients, n (%)	272 (60.9)		118 (48.6)		126 (73.3)				28 (87.5)			
		Number, mean (SD)	1.3 (1.5)		0.8 (1.1)		1.7 (1.6)				2.7 (2.1)			
	Cumulative OCS dose in mg equivalent prednisone, mean (SD)		240.5 (376.2)		131.3 (223.2)		337.2 (414.3)				550.4 (675.3)		<.001	
	**Respiratory antibiotics**												<.001	
		Patients, n (%)	336 (75.2)		177 (72.8)		132 (76.7)				27 (84.4)			
		Number, mean (SD)	3.9 (4.9)		3.4 (5.1)		4.3 (4.4)				6.1 (5.3)			
	**ICS/R03^h^ ratio, n (%)**												<.001	
		0	56 (12.5)		37 (15.2)		16 (9.3)				3 (9.4)			
		<0.5	81 (18.1)		20 (8.2)		47 (27.3)				14 (43.8)			
		≥0.5	119 (26.6)		43 (17.7)		64 (37.2)				12 (37.5)			
		Not assessable^i^	191 (42.7)		143 (48.8)		45 (26.2)				3 (9.4)			

^a^ICS: inhaled corticosteroid.

^b^FDC: fixed-dose combination.

^c^LABA: long-acting beta agonist.

^d^N/A: not applicable.

^e^LTRA: leukotriene receptor antagonist.

^f^SABA: short-acting β2 agonist.

^g^OCS: oral corticosteroid.

^h^R03: group of medications used in the treatment of obstructive airway diseases (Anatomical Therapeutic Chemical classification).

^i^Patients not receiving any respiratory therapy.

### ICS Trajectories Before ARH in the Children

**Figure 3:**
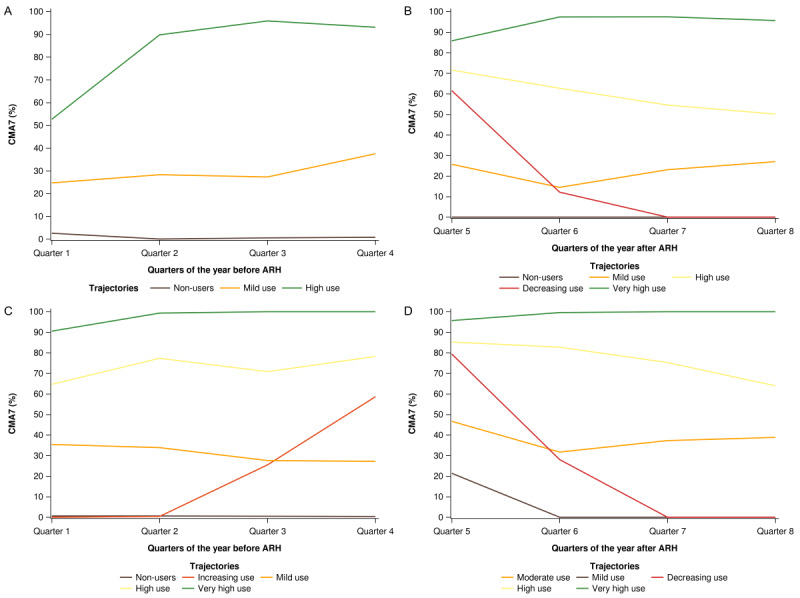
Inhaled corticosteroid trajectories of children (n=447) and teens/adults (n=416), before (A and C, respectively), and after (B and D, respectively) asthma-related hospitalization (ARH). CMA7: Continuous Measures of Medication Acquisition-7.

Three trajectories of ICS use were distinguished before ARH in the children: (1) no use (CMA7 value around 0%), (2) mild use (CMA7 value of 15%-40%), and (3) high use (CMA7 value of >80% during at least 3 quarters of the year before ARH) (Figure 3A). Just over half of the children (243/447, 54.4%) had a no-use typology, 38.5% (172/447) had a mild-use typology, and 7.2% (32/447) had a high-use typology during the last 3 quarters before ARH.

The no-use group comprised a larger percentage of males (167/243, 68.7%) compared to the mild-use (107/172, 62.2%) or high-use (17/32, 53.1%) group. There was extensive use of OCSs, with rates of 48.6% (118/243; MAD of 223 mg), 73.3% (126/172; MAD of 414 mg), and 87.5% (28/32; MAD of 675 mg) among the no-use, mild-use, and high-use groups, respectively. Respiratory antibiotics were dispensed to 72.8% (177/243), 76.7% (132/172), and 84.4% (27/32) of the children in the no-use, mild-use, and high-use groups, respectively (Table 1). The children in the mild-use (141/172, 82.0%) and high-use (29/32, 90.6%) groups had more visits to the respiratory physician or pediatrician than those in the no-use group (158/243, 65.0%).

### ICS Trajectories After ARH in the Children

In the year following ARH, 5 trajectories were distinguished in the children (Figure 3B). More than half of the children (239/447, 53.5%) had CMA7 values >50%. These included either very high users (n=67) with CMA7 values of >80% throughout the year or high users (n=172) with CMA7 values declining from 70% to 50% throughout the year. Nonusers (CMA7=0) corresponded to 15.0% (67/447) of all children. Mild users (n=78) had stable CMA7 values (20%-30%) throughout the following year, whereas decreasing users (n=63) had CMA7 values of 60% at the beginning of the year, which declined to zero by the end of the year.

### Changes in Trajectories (Before-After ARH) in the Children

**Figure 4:**
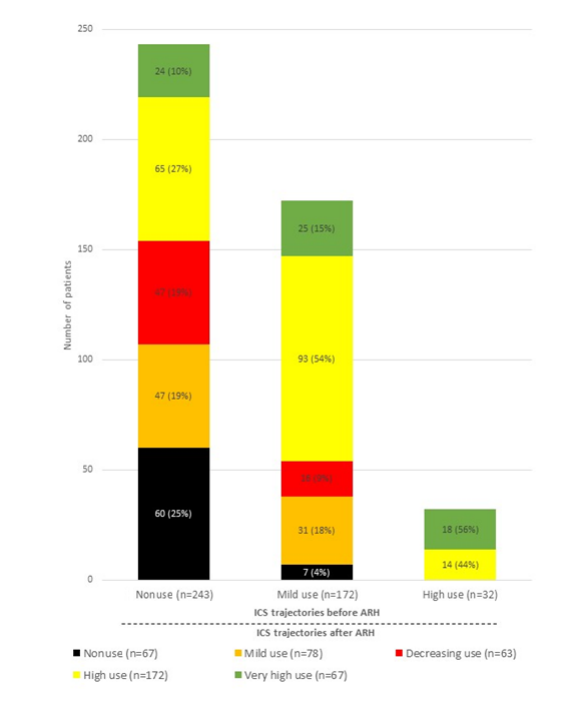
Distribution of inhaled corticosteroid (ICS) trajectories of children before and after asthma-related hospitalization (ARH) (n=447).

All children in the high-use group before ARH maintained a high-use profile during the year after hospitalization (Figure 4). Almost a quarter (60/243, 24.7%) of nonusers before ARH remained unchanged in the following year, whereas one-third (89/243, 36.6%) of nonusers switched to the high-use group after ARH. More than two-thirds (149/243, 61.3%) of mild users before ARH increased their use of ICSs after ARH, with 54.1% (93/172) becoming high users and 14.5% (25/172) becoming very high users. Graphs presenting the between-group interchange of individual patients are presented in Multimedia Appendix 2.

### Population Characteristics Before ARH in the Teens/Adults

At baseline, the mean age of the teens/adults was 45.7 years, and most patients were female (254/416, 61.1%). Moreover, 13.5% (56/416) had free access to care, 15.4% (64/416) had a chronic disease status for asthma, and 38.5% (160/416) had visited a respiratory physician or pediatrician. Almost one-third (137/416, 32.9%) of the patients had one or more ER visits, and 30.0% (125/416) underwent pulmonary function testing (Table 2). At the time of the study, 11.8% (49/416) of the teens/adults were using LABAs in monotherapy, 70.4% (293/416) received respiratory antibiotics, and 55.5% (231/416) received OCSs. Additionally, few patients (112/416, 26.9%) had high therapeutic ratios (≥0.5).

**Table 2:** Characteristics of the teens/adults at baseline (T0) overall and in the different preasthma-related hospitalization trajectories.

Characteristic			All (N=416)	No use (n=134)	Increasing use (n=45)	Mild use (n=100)	High use (n=82)	Very high use (n=55)	*P* value
**Sociodemographic characteristics**									
	**Gender, n (%)**								
		Male	162 (38.9)	56 (41.8)	18 (40.0)	36 (36.0)	27 (32.9)	25 (45.5)	.55
	Age at baseline (years), mean (SD)		45.7 (22.5)	37.5 (21.1)	44.0 (21.0)	44.6 (23.5)	54.0 (21.1)	56.8 (18.5)	
	**Age group, n (%)**								<.001
		11-35 years	148 (35.6)	75 (56.0)	15 (33.3)	36 (36.0)	16 (19.5)	6 (10.9)	
		36-50 years	88 (21.2)	24 (17.9)	14 (31.1)	20 (20.0)	17 (20.7)	13 (23.6)	
		51-70 years	110 (26.4)	22 (16.4)	10 (22.2)	24 (24.0)	30 (36.6)	24 (43.6)	
		≥71 years	70 (16.8)	13 (9.7)	6 (13.3)	20 (20.0)	19 (23.2)	12 (21.8)	
	Free access to care status, n (%)		56 (13.5)	14 (10.4)	7 (15.6)	22 (22.0)	6 (7.3)	7 (12.7)	.04
	Chronic disease status for asthma, n (%)		64 (15.4)	4 (3.0)	6 (13.3)	12 (12.0)	21 (25.6)	21 (38.2)	<.001
**Health care resource utilization before ARH^a^ (12 months)**									
	**Respiratory physician or pediatrician visits**								<.001
		Patients, n (%)	160 (38.5)	37 (27.6)	22 (48.9)	43 (43.0)	40 (48.8)	18 (32.7)	
		Number, mean (SD)	0.9 (1.8)	0.5 (1.2)	1.2 (2.1)	0.9 (1.6)	1.2 (1.8)	0.9 (3.0)	
	**Pulmonary function testing**								<.001
		Patients, n (%)	125 (30.0)	7 (5.2)	15 (33.3)	30 (30.0)	39 (47.6)	34 (61.8)	
		Number, mean (SD)	0.6 (1.2)	0.1 (0.4)	0.7 (1.5)	0.5 (1.1)	0.9 (1.3)	1.6 (1.7)	
	**ER^b^ visits**								.19
		Patients, n (%)	137 (32.9)	43 (32.1)	14 (31.1)	42 (42.0)	26 (31.7)	12 (21.8)	
		Number, mean (SD)	0.6 (1.2)	0.5 (1.0)	0.6 (1.8)	0.8 (1.5)	0.5 (1.0)	0.3 (0.7)	
	**ARH**								.89
		Patients, n (%)	12 (2.9)	3 (2.2)	2 (4.4)	2 (2.0)	3 (3.7)	2 (3.6)	
		Number, mean (SD)	0.1 (0.4)	0.1 (0.5)	0.0 (0.2)	0.0 (0.1)	0.0 (0.2)	0.1 (0.8)	
**Asthma therapy**									
	**ICSs^c^**								<.001
		Patients, n (%)	130 (31.3)	19 (14.2)	12 (26.7)	40 (40.0)	32 (39.0)	27 (49.1)	
		Number, mean (SD)	1.3 (3.6)	0.3 (1.0)	0.7 (1.7)	1.3 (2.5)	1.6 (2.9)	3.7 (7.8)	
	**FDCs^d^ of ICSs/LABAs^e^**								<.001
		Patients, n (%)	212 (51.0)	24 (17.9)	21 (46.7)	60 (60.0)	61 (74.4)	46 (83.6)	
		Number, mean (SD)	3.4 (5.3)	0.3 (0.8)	1.4 (2.0)	2.4 (2.8)	5.7 (5.2)	10.9 (7.9)	
	**LABAs (in a separate canister)**								.05
		Patients, n (%)	49 (11.8)	7 (5.2)	6 (13.3)	15 (15.0)	11 (13.4)	10 (18.2)	
		Number, mean (SD)	0.9 (3.3)	0.4 (2.0)	1.2 (3.9)	0.7 (2.2)	0.8 (2.7)	2.6 (6.2)	
	**LTRAs^f^**								<.001
		Patients, n (%)	104 (25.0)	7 (5.2)	11 (24.4)	23 (23.0)	32 (39.0)	31 (56.4)	
		Number, mean (SD)	1.7 (3.8)	0.3 (1.8)	1.6 (3.5)	1.2 (2.9)	2.2 (3.9)	5.6 (5.9)	
	**SABAs^g^**								<.001
		Patients, n (%)	259 (62.3)	49 (36.6)	25 (55.6)	79 (79.0)	62 (75.6)	44 (80.0)	
		Number, mean (SD)	3.9 (6.9)	1.0 (2.3)	4.5 (7.8)	3.5 (3.6)	4.6 (5.8)	9.9 (13.3)	
	**Respiratory antibiotics**								<.001
		Patients, n (%)	293 (70.4)	86 (64.2)	28 (62.2)	71 (71.0)	58 (70.7)	50 (90.9)	
		Number, mean (SD)	5.0 (7.6)	2.8 (3.6)	3.6 (5.5)	4.6 (7.1)	6.3 (7.9)	9.7 (12.9)	
	**OCSs^h^**								<.001
		Patients, n (%)	231 (55.5)	58 (43.3)	26 (57.8)	60 (60.0)	54 (65.9)	33 (60.0)	
		Number, mean (SD)	2.4 (5.8)	1.2 (3.5)	2.9 (5.5)	1.9 (3.2)	3.7 (9.1)	4.0 (7.4)	
	Cumulative OCS dose in mg equivalent prednisone, mean (SD)		695.5 (1319.4)	350.5 (703.5)	806.3 (1476.6)	702.2 (1072.3)	797.1 (1163.5)	1281.9 (2360.4)	<.001
	**ICS/R03^i^ ratio, n (%)**								<.001
		0	44 (10.6)	28 (20.9)	6 (13.3)	8 (8.0)	2 (2.4)	0 (0.0)	
		<0.5	168 (40.4)	19 (14.2)	17 (37.8)	50 (50.0)	42 (51.2)	40 (72.7)	
		≥0.5	112 (26.9)	19 (14.2)	9 (20.0)	35 (35.0)	36 (43.9)	13 (23.6)	
		Not assessable^j^	92 (22.1)	68 (50.7)	13 (28.9)	7 (7.0)	2 (2.4)	2 (3.6)	

^a^ARH: asthma-related hospitalization.

^b^ER: emergency room.

^c^ICS: inhaled corticosteroid.

^d^FDC: fixed-dose combination.

^e^LABA: long-acting beta agonist.

^f^LTRA: leukotriene receptor antagonist.

^g^SABA: short-acting β2 agonist.

^h^OCS: oral corticosteroid.

^i^R03: group of medications used in the treatment of obstructive airway diseases (Anatomical Therapeutic Chemical classification).

^j^Patients not receiving any respiratory therapy.

### ICS Trajectories Before ARH in the Teens/Adults

Among the teens/adults, 5 ICS trajectories were distinguished before ARH. These were as follows: (1) no use (CMA7 value of about 0; 134/416, 32.2%), (2) mild use (CMA7 value of 30%-35%; 100/416, 24.0%), (3) increasing use over time (CMA7 value rising from 0% 1 year before ARH to 60% at the time of ARH; 45/416, 10.8%), (4) high use (CMA7 value of 60%-80%; 82/416, 19.7%), and (5) very high use (CMA7 value of >90%; 55/416, 13.2%) (Figure 3C). More than half of nonusers (75/134, 56.0%) were aged between 11 and 35 years, whereas almost two-thirds of high (49/82, 59.8%) or very high (36/55, 65.5%) users were older than 50 years. The sex ratios also differed between groups, with males representing 41.8% (56/134) of nonusers versus 32.9% (27/82) of high users (Table 2). A chronic disease status for asthma was more frequent among high and very high users (21/82, 25.6% and 21/55, 38.2%, respectively) than among nonusers (4/134, 3.0%). Nonusers and very high users had fewer visits to a respiratory physician (37/134, 27.6% and 18/55, 32.7%, respectively) compared with the other groups (>40%). In terms of asthma therapy, very high users were frequently treated with FDCs, LABAs (in monotherapy), SABAs (10 units per year), respiratory antibiotics (91% of users receiving 10 units per year on average), and OCSs (total annual dose of 1282 mg), while their therapeutic ratios were low overall (40/55, 72.7%).

### ICS Trajectories After ARH in the Teens/Adults

These patients were also distributed across 5 groups in the year following ARH. Half of them (209/416, 50.2%) were very high (CMA7 value of >90%) or high (CMA7 value of >65%) users, whereas 44.7% (186/416) were mild (CMA7 value of <20%) or moderate (CMA7 value of 30%-50%) users. The last group included a few patients (21/416, 5.1%) with decreasing use during the year following ARH, from 80% shortly after ARH to zero after the third quarter of the subsequent year (Figure 3D).

### Changes in Trajectories (Before-After ARH) in the Teens/Adults

**Figure 5:**
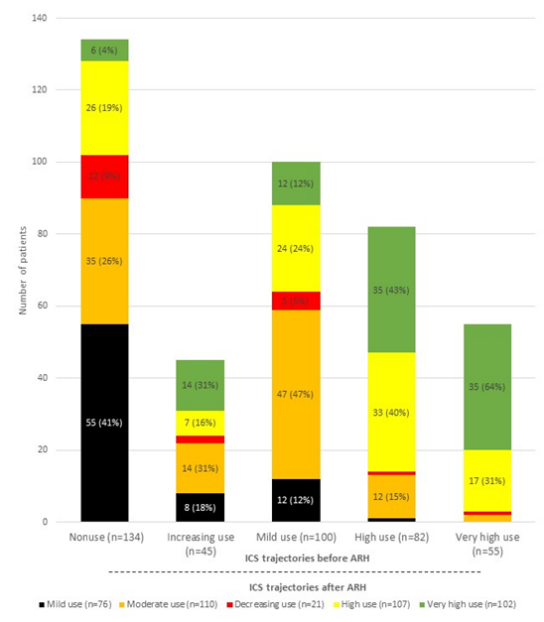
Distribution of inhaled corticosteroid (ICS) trajectories of teens/adults before and after asthma-related hospitalization (ARH) (n=416).

While 23.9% (99/416) of nonusers before ARH started to use ICS regularly after ARH, most patients in that group remained in an irregular use profile in the year following their hospital stay, with 41.0% (55/134) in the mild use group and 9.0% (12/134) in the decreasing use group (Figure 5). Another quarter (32/134, 26.1%) of nonusers increased their use constantly throughout the subsequent year and were thus transferred to the moderate use group.

In the group with increasing ICS use before ARH, 31.1% (14/45) stayed at a similar CMA7 level and 31.1% (14/45) switched to the very-high-use group. Lastly, most patients in the other 3 groups (mild, high, and very high use) exhibited a similar profile of ICS use after ARH compared with the year prior to ARH. Patients with CMA7 values of >60% either stayed in the same profile group or switched to the closest similar profile group (high-use to very-high-use group at 42.7% [35/82] and very-high-use to high-use group at 30.9% [17/55]). Patients in the mild-use group before ARH mostly stayed in this profile group, while 47.0% (47/100) of them joined the moderate-use group during the year after ARH. The overall trajectories of the teen/adult patients switching from groups are presented in Multimedia Appendix 3.

## Discussion

Our study of the long-term routine care of pediatric and adult patients admitted to the hospital for asthma exacerbation (ARH) showed major temporal changes in ICS use before and after ARH. ARH seems to have similar effects on the typology of use in children and teens/adults. Indeed, in both populations, segmented regression analyses showed 3 contrasting segments: pre-ARH segment (Q1-Q3) with stable use, ARH segment (Q4-Q5) with increasing use, and post-ARH segment (Q6-Q8) with use decreasing again. Interestingly, for children, the post-ARH levels of ICS use did not appear to decline to the pre-ARH levels. As a rule, trajectories were related to patient sociodemographic characteristics, health care pathways, and asthma therapy, in the context of overall poor quality of care.

Overall, our study identified 3 sets of behaviors regarding the impact of ARH on ICS use. First, in terms of improved typology, 46.3% (207/447) of nonusers or mild users before ARH increased their ICS use after hospital stay. This was the case for 46.5% (208/447) of children and 39.9% (166/416) of teens/adults with low ICS use before ARH. This may correspond to a recent or reconfirmed diagnosis of asthma.

Second, in terms of the limited impact of ARH on typology, most regular users (high-use and very-high-use groups) before ARH remained in the high or very-high-use group after ARH. These patients likely had been identified as experiencing more severe or difficult asthma before ARH and remained regularly treated. This assumption is supported by patient characteristics, such as a high rate of a chronic disease status, which is typically allocated to more severe patients, in addition to a high proportion of OCS users.

Lastly, in terms of decreased use, a minority of patients from the high-use or very-high-use group shifted to the decreasing-use or low-use group.

In all trajectories, a majority of the children had visited a pediatrician or a respiratory physician, and many children had visited the ER, suggesting that asthma was suspected before ARH, despite the low age of the pediatric sample population [21]. The use of OCSs and respiratory antibiotics was also very common before ARH, with a large majority of patients receiving respiratory antibiotics and OCSs among high ICS users. In this group, the annual dose of OCSs far exceeded the dose considered to be the threshold for OCS severe side effects [22]. The therapeutic ratio in this group was also of concern since almost half (14/32, 44%) of the children had a low ratio during the baseline period, implying an overuse of SABAs [23]. Overall, the data may reflect severity, but they do also suggest an overall poor quality of asthma care in the pediatric population.

Among teens/adults, the ratios were low (<0.5) in many teens/adults in the baseline period, which again reflects inappropriate care of asthma. The use of OCSs was also high in all teens/adults, and was particularly high (MAD of >1000 mg) in the very-high-use group, in addition to very high use of SABAs and respiratory antibiotics. More than one in 10 teens/adults inappropriately received LABAs in a separate canister [24].

Overall, the data show that although the clustering-derived typologies differed between children and teens/adults in the pre-ARH period, the global trajectories of these 2 independent samples were similar, supporting the validity of our results. A major finding is that ARH occurred in the context of low quality of care and overuse of medical resources in a population largely affected by social deprivation, as already pointed out by prior studies [25]. Our study does indeed illustrate the high proportion of patients visiting ERs before ARH (more than one-third of children [171/447, 38.3%] and teens/adults [137/416, 32.9%]), the low therapeutic ratios, and the extensive use of OCSs and respiratory antibiotics.

Regarding existing literature, studies assessing ICS use in asthma over an extended period are scarce [26]. This is particularly the case outside the context of specific prospective cohorts and thus limits the comparison with other data. In a prospective study, Krishnan et al [9] showed a rapid decrease in the use of ICSs and OCSs by asthmatic patients after hospital discharge, but no information was provided on their use before admission. Another study showed inappropriate use in children after ARH; however, again, there was no assessment of their use before ARH [27]. Similarly, Williams et al [10] clarified the overall relationship between changing ICS use and the occurrence of severe exacerbations, including ARH. Prior data on the relationship between ICS use and exacerbations may however not be generalizable since asthmatics are heterogeneous in their use of therapy and their behaviors may differ in routine care compared to investigational conditions. Of interest, a recent Danish study investigated the effect of hospital admissions for acute exacerbation on the adherence rate to controller medication in 241 adults with asthma. Their results were similar to ours in that an initial improvement in adherence to ICSs after hospital admission occurred in patients who had previously shown poor adherence. The study also showed that the improvement was transient and decreased over time during the 6 months after discharge [28]. International comparisons are however complex since patient behaviors toward therapy also depend on the local organization of health care with, for instance, the level of care co-payments, or the accessibility to health care professionals, which may affect the relationship between ICS use and exacerbation. This underlines the need to replicate investigations in different settings.

Our study had several strengths. It was a population-based study, and it was conducted with a random sample originating from data compiled from the health care consumption information of almost all French citizens (over 67 million inhabitants), without distinction of age, gender, ethnicity, residency, income, and social status, in a large country with a single universal health care system. These strengths allowed us to overcome the limitations of other studies on asthma care, such as distinct health care coverage for different patient groups or missing information as a result of fragmented care [29].

The study also had some limitations. There was no information on the education level of the patients and whether this had an impact on their trajectory. There was also no information on smoking status, pulmonary function, or BMI that may have impacted the effects of ICSs. We used CMA7 to assess ICS use. Considering that claims information provides medicine dispensing data, CMA7 measures were based on the dispensing number and frequency but did not reflect actual medication use or provide data on the quality of the inhaler technique. Additionally, we included all patients undergoing ARH during the study period independent of their ICS use and asthma diagnosis before ARH. As an alternative, we could have restricted the inclusion to patients who received an initial diagnosis of asthma during their hospital stay. Such cases were however rare among both children and teens/adults, as evidenced by the high percentage of visits to a respiratory specialist (115/447, 25.7% and 160/416, 38.5%, respectively), and by the common records of pulmonary function tests performed during the baseline period. Consequently, the impact on the results should be limited. Another limitation may have been due to the exclusion of patients who did not have continuous follow-up information recorded in the database either before or after ARH. However, those patients were excluded for administrative reasons, that is, they were affiliated to rare insurance schemes not included in the database. We feel confident that there is no reason why those patients would differ in their typologies compared with the patients included in the cohort. Finally, it may be of interest to reproduce a similar analysis in a larger population, as our sample was limited to 447 children and 416 teens/adults.

Overall, our study demonstrated inappropriate ICS use in routine care before ARH, which transiently improved shortly before and after discharge in the context of overall poor quality of care, in a largely socially deprived population experiencing persistent asthma. Our results suggest that interventions are urgently needed to improve the use of controller therapy for asthma in routine care [30], particularly during or after ARH [31]. These interventions should be based on specific patient profiles and trajectories of use before ARH. For instance, therapeutic education with action plans [32,33] or the setup of maintenance and relief therapy [34] could be a priority for some trajectories. As a rule, clinicians should emphasize the need to make regular use of controllers at appropriate doses and to make parsimonious use of relievers and OCSs. The findings are consistent with those of a recent study on care for persistent asthma that showed a decrease in primary care physician involvement and an increase in the incidence of ER visits between 2006 and 2016 [35].

In conclusion, we showed that although ARH had an overall positive impact on the trajectories of ICS use, this effect was often transient, patient behaviors were heterogeneous, and several distinctly different trajectories were identified. Additional patient and care characteristics could further improve the understanding of our findings and help more efficiently target patients in need of preventive interventions. Our data reinforce the evidence that inappropriate use of ICSs paves the way for ARH, and thus, the findings should galvanize health service efforts to keep individual ICS use at optimal levels.

## Appendix

Multimedia Appendix 1 Study design.

Multimedia Appendix 2 Use of inhaled corticosteroid trajectories before and after asthma-related hospitalization by group before asthma-related hospitalization among children (n=447).

Multimedia Appendix 3 Use of inhaled corticosteroid trajectories before and after asthma-related hospitalization by group before asthma-related hospitalization among teens/adults (n=416).

## Abbreviations

ARH: asthma-related hospitalization
CMA7: Continuous Measures of Medication Acquisition-7
EGB: Echantillon Généraliste des Bénéficiaires (General Sample of Beneficiaries)
ER: emergency room
FDC: fixed-dose combination
ICD-10: International Classification of Diseases, 10th Revision
ICS: inhaled corticosteroid
LABA: long-acting beta agonist
LTRA: leukotriene receptor antagonist
MAD: mean annual dose
OCS: oral corticosteroid
SABA: short-acting β2 agonist

## Data Availability

Owing to National Health Service and Système National des Données de Santé (National Health Data System) rules, no data sharing is possible, as access to data is restricted to habilitated and qualified researchers (MN and FJ are habilitated and qualified).
