# Supplementary figures and images for: Trajectories of Controller Therapy Use Before and After Asthma-Related Hospitalization in Children and Adults: Population-Based Retrospective Cohort Study
Source: JMIR Public Health Surveill. 2023 Sep 26;9:e50085. doi: 10.2196/50085 (PMC10565628; doi:10.2196/50085)

## Multimedia Appendix 1. Study design.

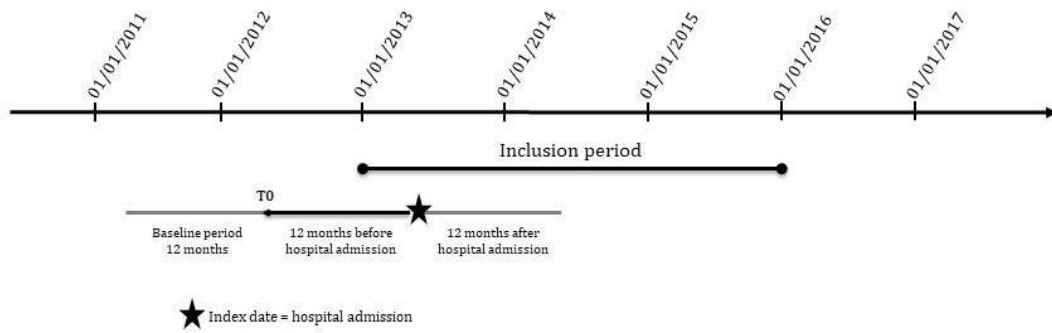

Supplement: Multimedia Appendix 1 [file publichealth_v9i1e50085_app1.pdf]
