# Supplementary material for: Trajectories of Controller Therapy Use Before and After Asthma-Related Hospitalization in Children and Adults: Population-Based Retrospective Cohort Study
Source: JMIR Public Health Surveill. 2023 Sep 26;9:e50085. doi: 10.2196/50085 (PMC10565628; doi:10.2196/50085)
Supplement: Multimedia Appendix 2 [file publichealth_v9i1e50085_app2.pdf]

Multimedia Appendix 2. Use of inhaled corticosteroid trajectories before and after asthma-related hospitalization by group before asthma-related hospitalization among children (n=447).

<u>Group before hospitalisation: non-users</u>

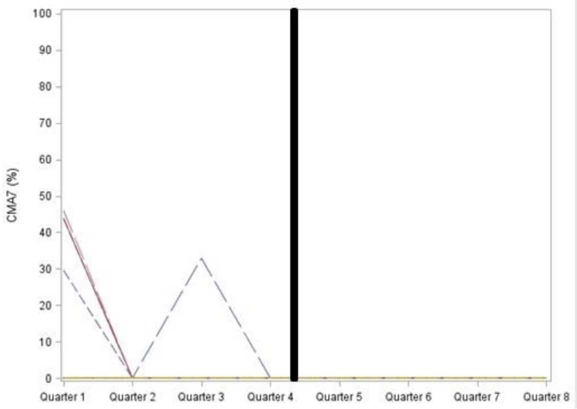

From no use to no use

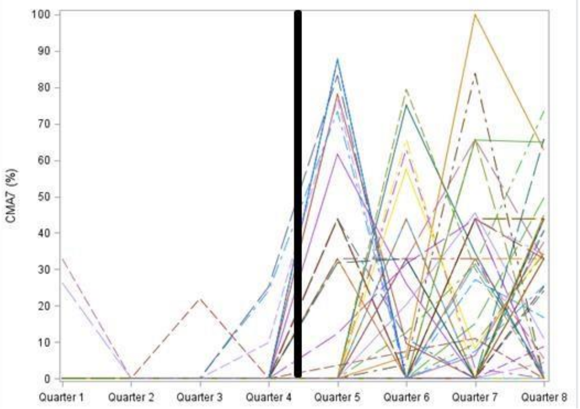

From no use to mild use

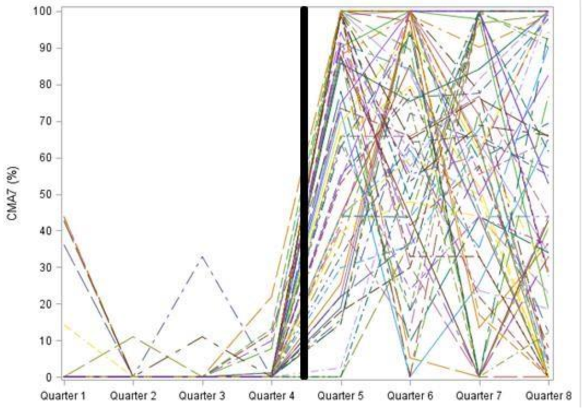

From no use to high use

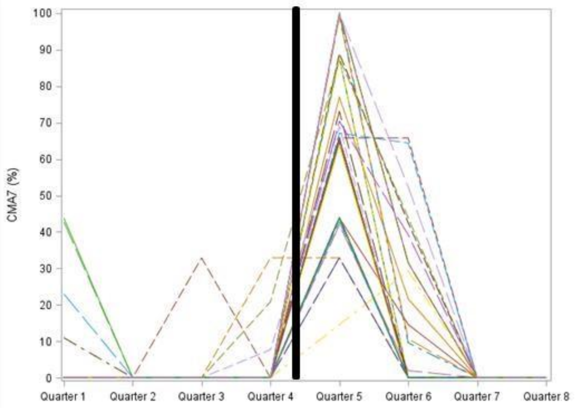

From no use to decreasing use

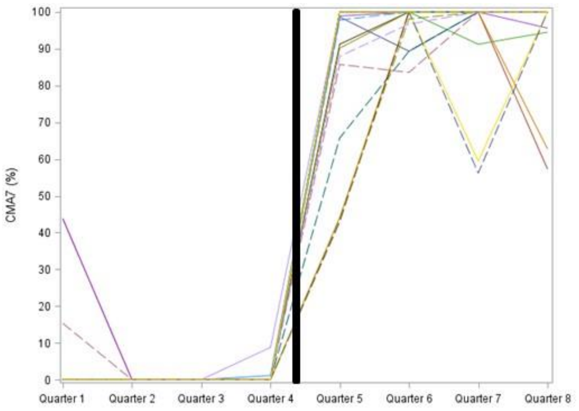

From no use to very high use

<u>Group before hospitalisation: mild use</u>

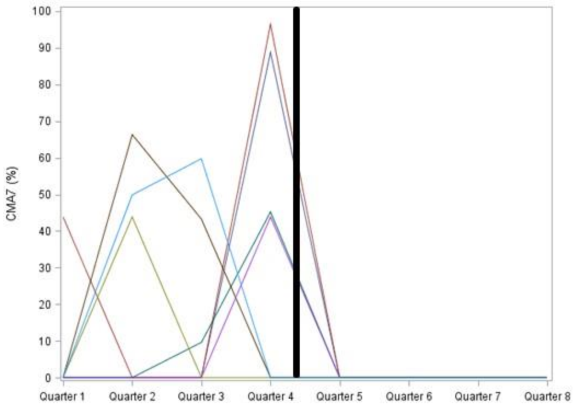

From mild use to no use

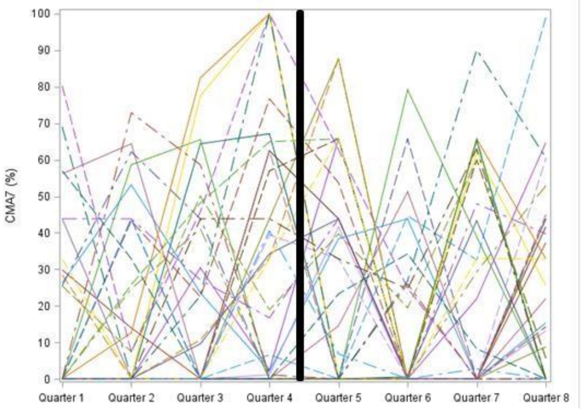

From mild use to mild use

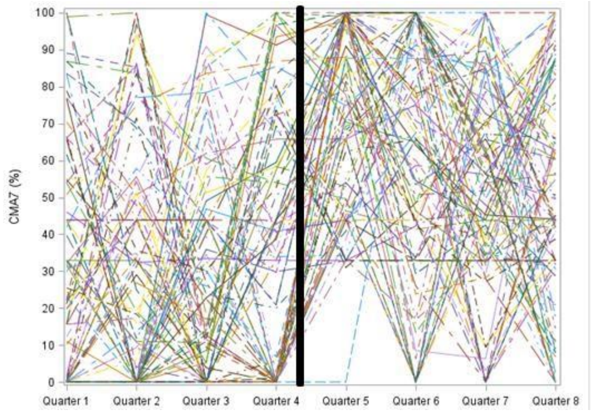

From mild use to high use

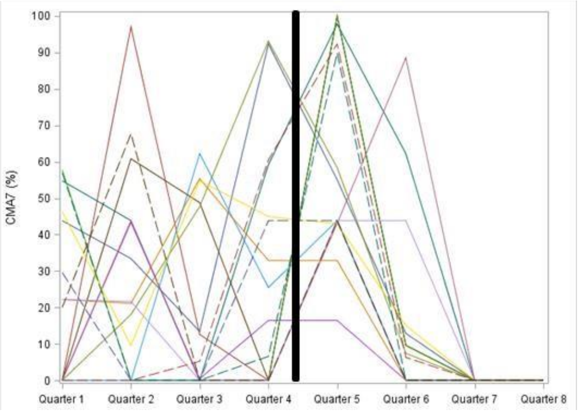

From mild use to decreasing use

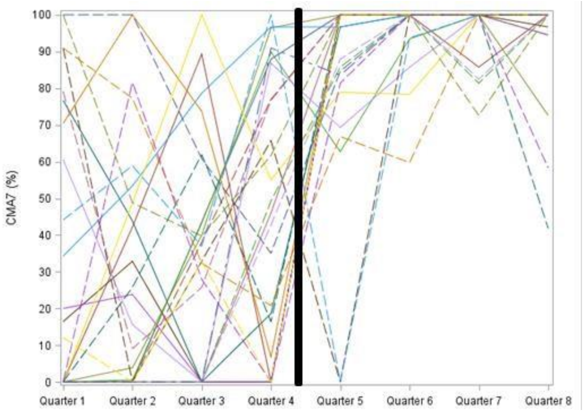

From mild use to very high use

<u>Group before hospitalisation: high use</u>

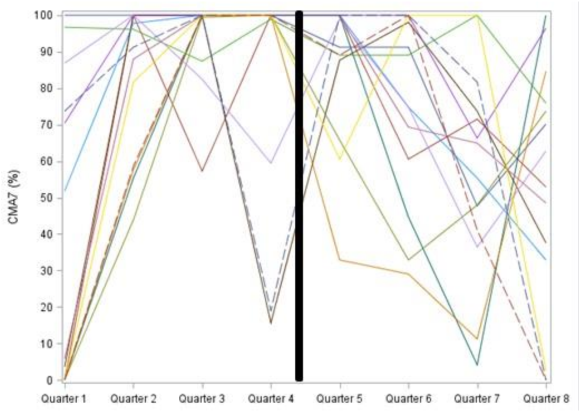

From high use to high use

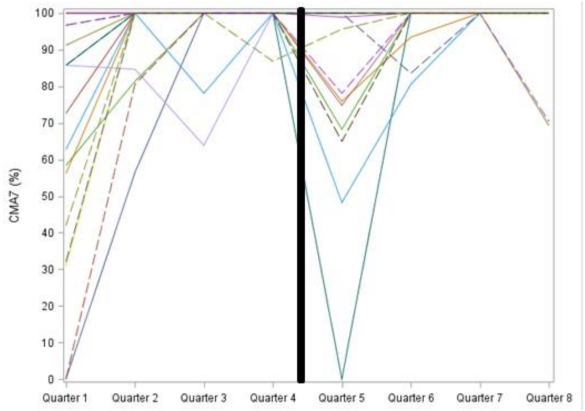

From high use to very high use

No patient switched from high use to no use, decreasing use or mild use, so there are only 2 graphs in this group.

Note: In these graphs, one line represents one patient
