# Supplementary material for: Trajectories of Controller Therapy Use Before and After Asthma-Related Hospitalization in Children and Adults: Population-Based Retrospective Cohort Study
Source: JMIR Public Health Surveill. 2023 Sep 26;9:e50085. doi: 10.2196/50085 (PMC10565628; doi:10.2196/50085)
Supplement: Multimedia Appendix 3 [file publichealth_v9i1e50085_app3.pdf]

**Multimedia Appendix 3.** Use of inhaled corticosteroid trajectories before and after asthma-related hospitalization by group before asthma-related hospitalization among teens/adults (n=416).

<u>Group before hospitalisation: no use</u>

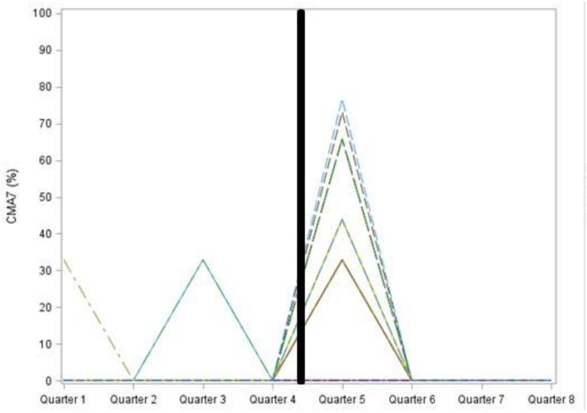


From no use to mild use

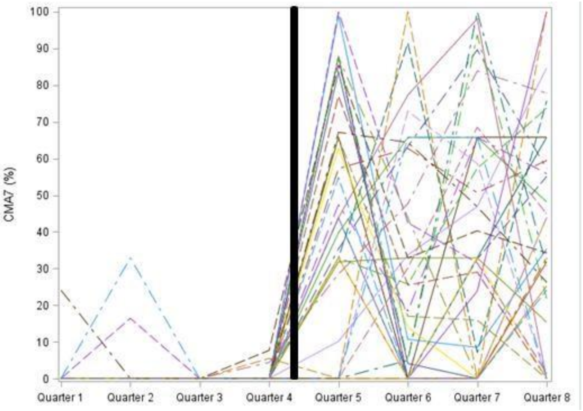


From no use to moderate use

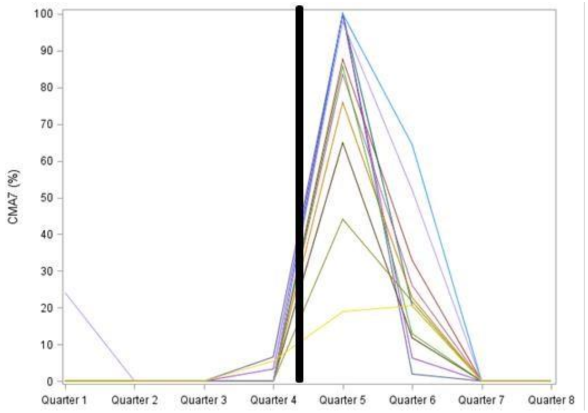


From no use to decreasing use

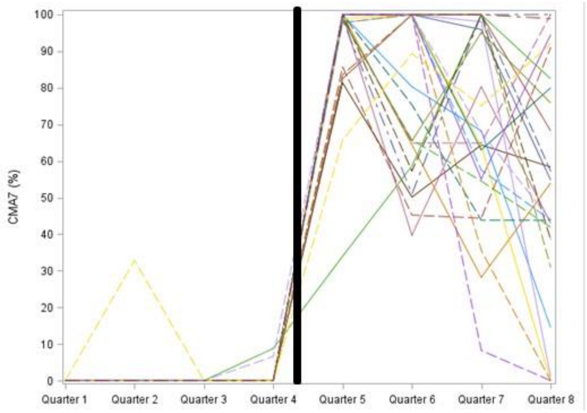


From no use to high use

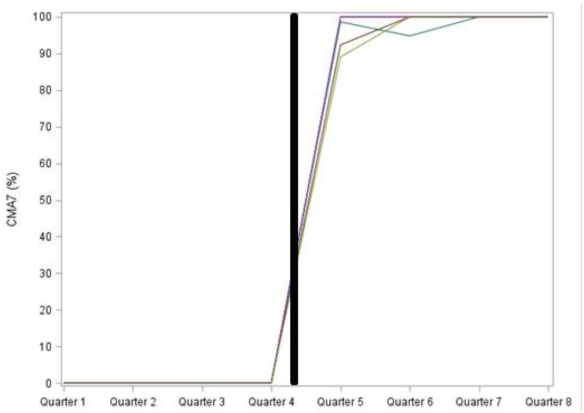


From no use to very high use

<u>Group before hospitalisation: increasing use</u>

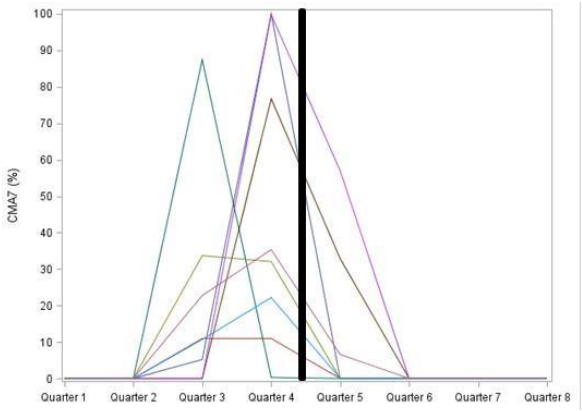


From increasing use to mild use

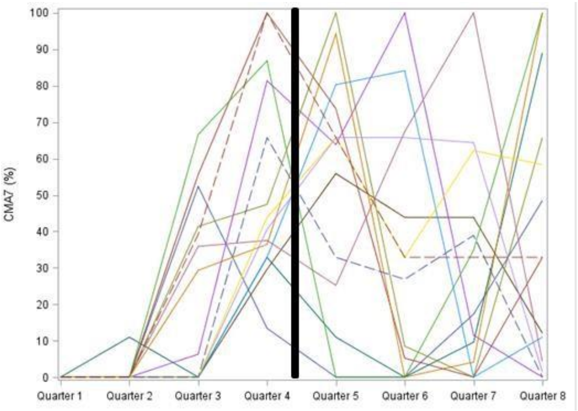


From increasing use to moderate use

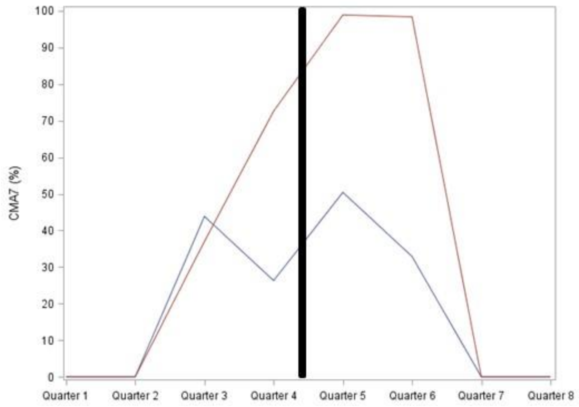


From increasing use to decreasing use

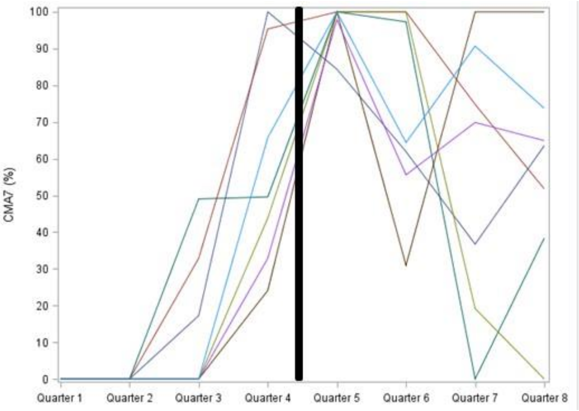


From increasing use to high use

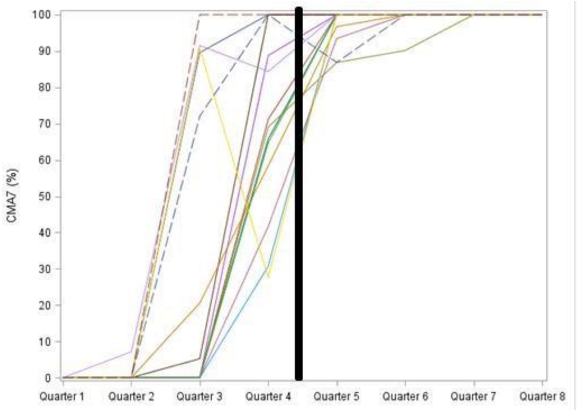


From increasing use to very high use

<u>Group before hospitalisation: mild use</u>

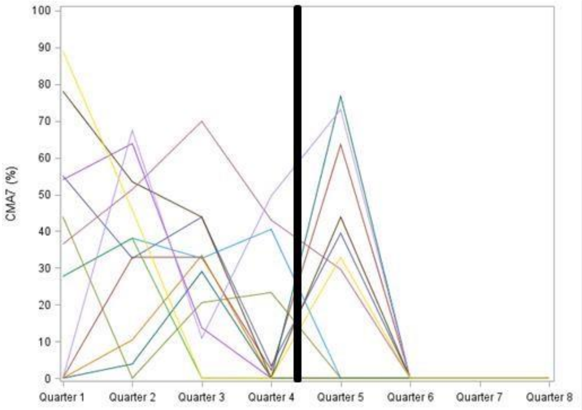


From mild use to mild use

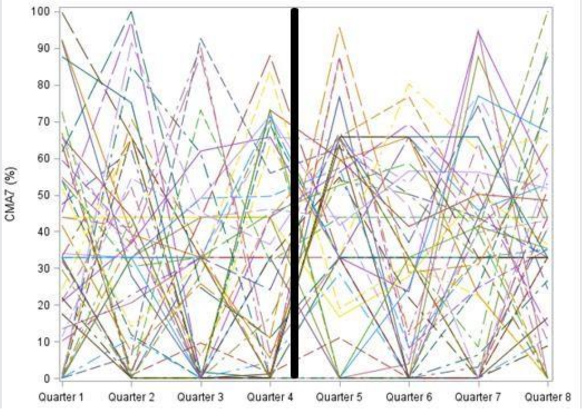


From mild use to moderate use

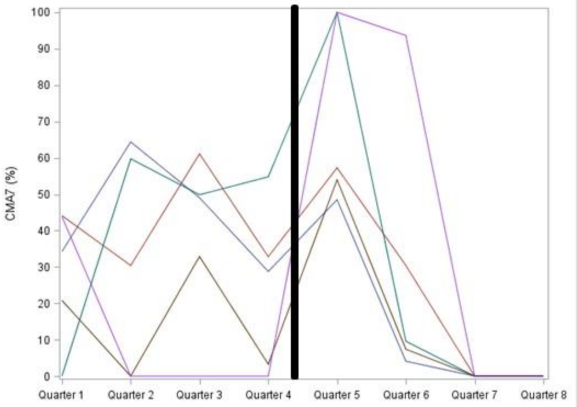


From mild use to decreasing use

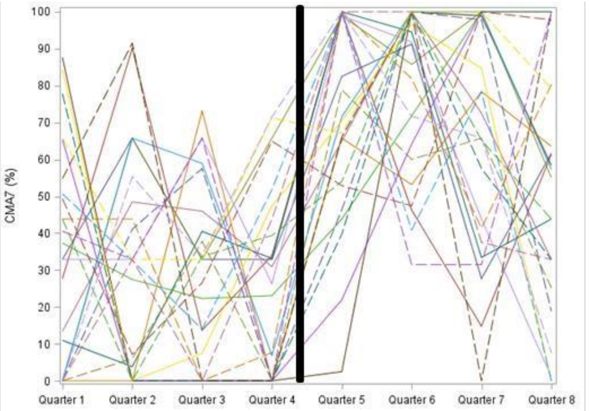


From mild use to high use

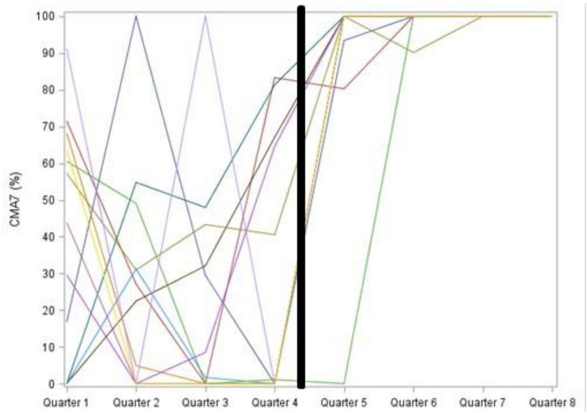


From mild use to very high use

<u>Group before hospitalisation: high use</u>

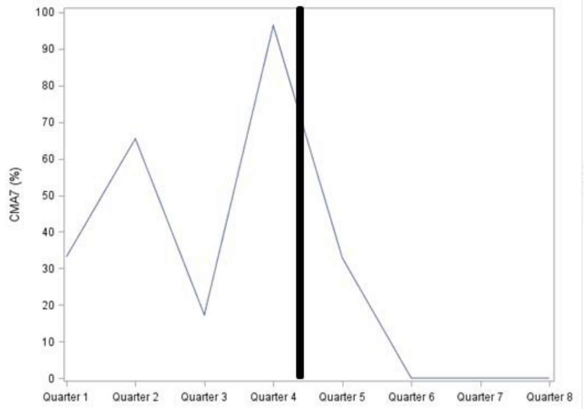


From high use to mild use

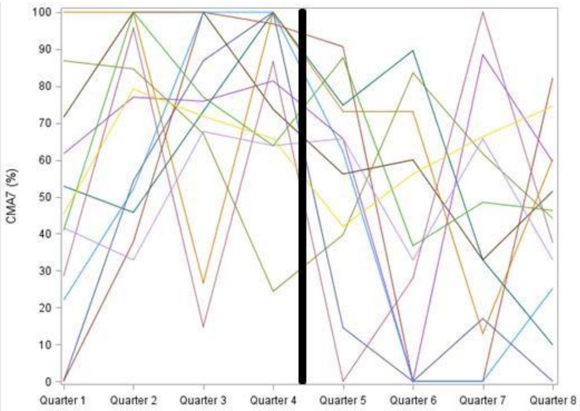


From high use to moderate use

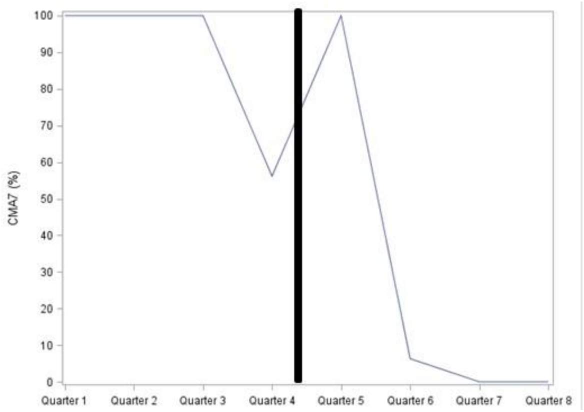


From high use to decreasing use

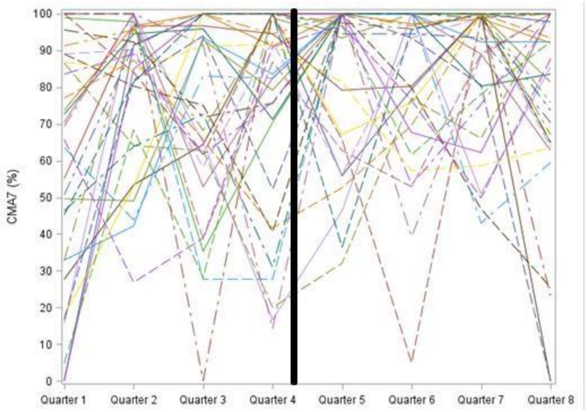


From high use to high use

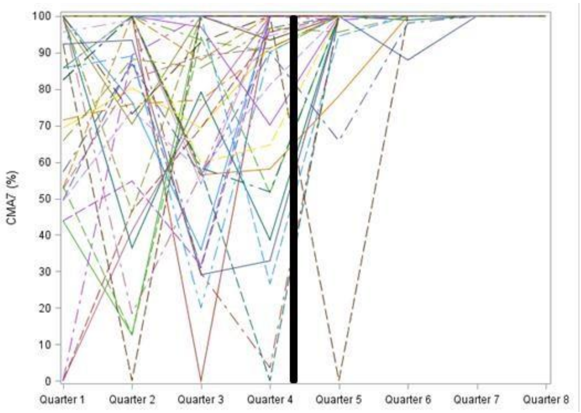


From high use to very high use

<u>Group before hospitalisation: very high use</u>

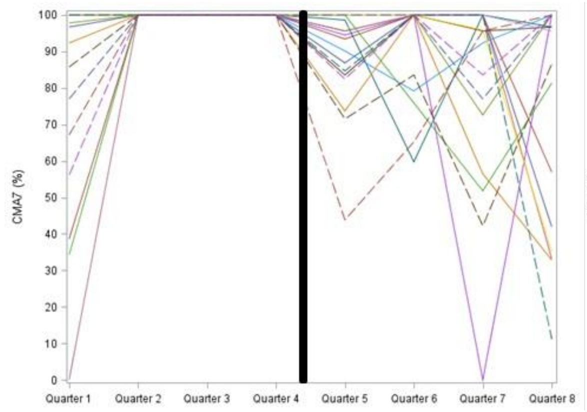


From very high use to high use

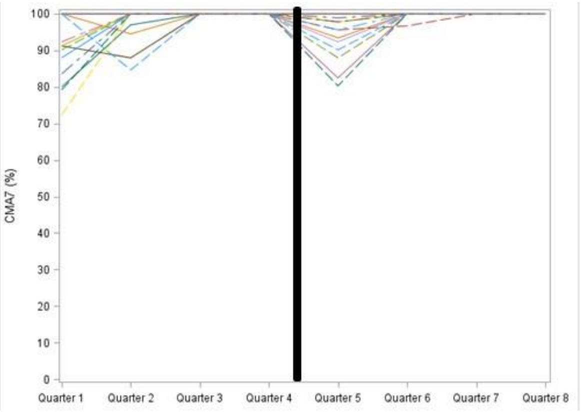


From very high use to very high use

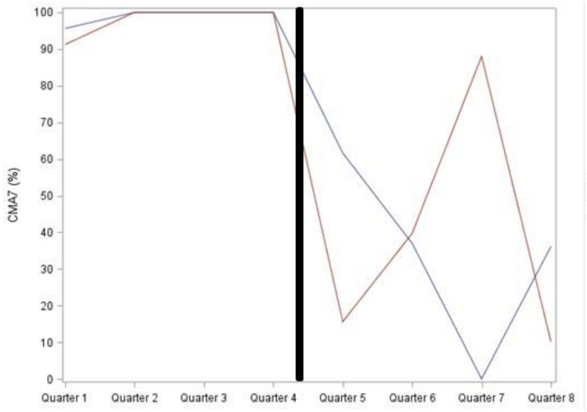


From very high use to moderate use

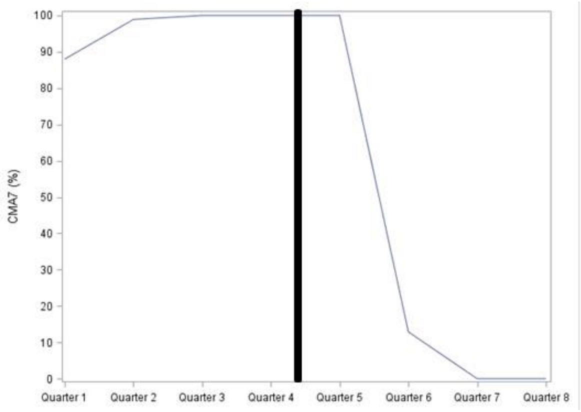


From very high use to decreasing use

No patient switched from very high use to mild use, so there are only 4 graphs in this group.

*Note: In these graphs, one line represents one patient*
